# Ethics of allocation of donor organs

**DOI:** 10.1097/MOT.0000000000001058

**Published:** 2023-02-10

**Authors:** Eline M. Bunnik

**Affiliations:** Department of Medical Ethics, Philosophy and History of Medicine, Erasmus MC, University Medical Centre Rotterdam, Rotterdam, The Netherlands

**Keywords:** allocation, donor organs, ethics, public preferences

## Abstract

There is no widely accepted single ethical principle for the fair allocation of scarce donor organs for transplantation. Although most allocation systems use combinations of allocation principles, there is a particular tension between ‘prioritizing the worst-off’ and ‘maximizing total benefits’. It is often suggested that empirical research on public preferences should help solve the dilemma between equity and efficiency in allocation policy-making.

**Recent findings:**

This review shows that the evidence on public preferences for allocation principles is limited, and that the normative role of public preferences in donor organ allocation policy making is unclear. The review seeks to clarify the ethical dilemma to the transplant community, and draws attention to recent attempts at balancing and rank-ordering of allocation principles.

**Summary:**

This review suggests that policy makers should make explicit the relative weights attributed to equity and efficiency considerations in allocation policies, and monitor the effects of policy changes on important ethics outcomes, including equitable access among patient groups. Also, it draws attention to wider justice issues associated not with the distribution of donor organs among patients on waiting lists, but with barriers in referral for transplant evaluation and disparities among patient groups in access to waiting lists.

## INTRODUCTION

Organ transplantation stands out as one of the areas in medicine that has the power to return patients with life-threatening illness to health and normal functioning within society. However, the practice of organ transplantation is overshadowed by an abiding shortage of suitable donor organs. There are approximately 14 000 patients on the waiting list for organ transplantation in the Eurotransplant region [1], and over 110 000 patients in the United States of America [2,3]. In the USA, 17 people die each day waiting for an organ transplant. This is a merciless consequence of the unceasing absolute scarcity of donor organs derived from deceased donors. Although allocation is inevitable, it is notoriously difficult to determine how it can be done fairly. There is no widely accepted single ethical principle for the just allocation of scarce medical resources [4].

Thus, most allocation systems use combinations of allocation principles [5]. Historically, systems have distributed organs based on severity and/or urgency of the disease, thus to patients with the highest mortality risk if left untreated, but also on the basis of time spent on the waiting list, and geographical region [6], combining logistical considerations with the ethical principles of ‘sickest first’ and ‘first-come, first served’. Ethicists, however, tend to find fault with these principles [4]. Although ‘first-come, first served’ may *seem* fair, they argue, it is not, because those who come first are often more health literature and assertive, and of higher socioeconomic status, than those who come later. Since the publication of the US Final Rule in 1999 [7], allocation systems have tried to minimize the role of waiting time, in favour of medical urgency. The ‘sickest first’ principle, however, is criticized, too, because it disregards posttransplant prognosis and might lead to worse health outcomes for all. Clearly, the discussion on the ethics of allocation of donor organs has not been settled. Recent scholarship suggests that empirical evidence on public preferences should be used to adapt or refine allocation systems [8^▪^]. How can such evidence be used sensibly in policy-making for the allocation of donor organs? 

**Box 1:**
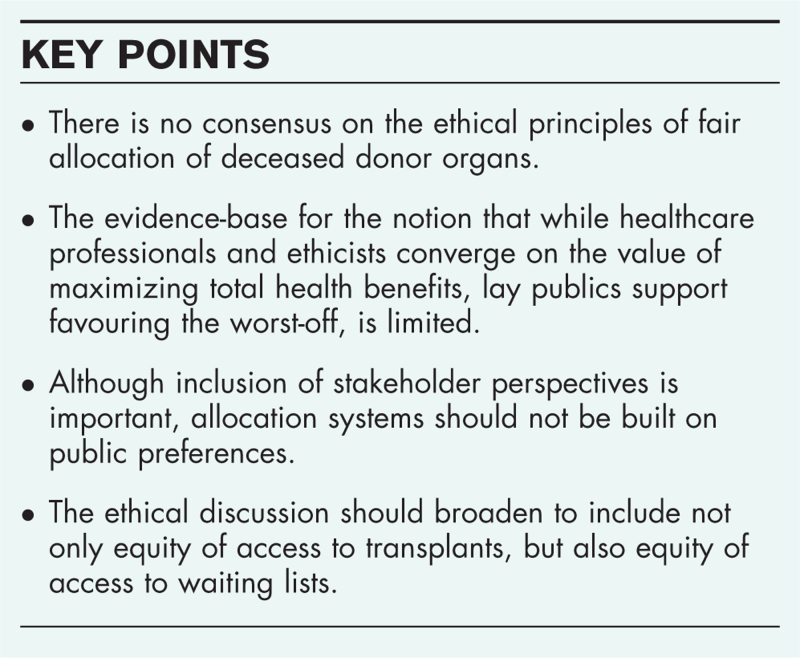
no caption available

## UNDERSTANDING THE ETHICAL DILEMMA

There are numerous allocation principles in circulation that reach for different aims. The ethical literate subdivides these principles into four categories: treating people equally, favouring the worst-off, maximizing total benefits and promoting and rewarding social usefulness [4]. The latter principle – promoting social usefulness – can be useful in pandemic settings, for example for priority distribution of vaccines and personal protective equipment to front-line healthcare workers, in order to maintain basic functioning of society [9^▪^]. In the context of organ transplantation, this principle is problematic, as all human beings are deemed to be of equal moral worth. The first principle – treating people equally – is a formal justice requirement, connected to ‘respect for persons’, one of the US Organ Procurement Transplantation Network (OPTN) ethical principles [10]. Allocation decisions should not be based on morally irrelevant criteria, such as sex, ethnicity, socioeconomic status, geographical location, etc. Thus understood, treating patients equally can be considered to serve as a ‘guardrail’ [11] or a *sine qua non* for any fair allocation system. Thus, the ethical discussion converges on the two remaining principles of ‘favoring the worst-off’ and ‘maximizing total benefits’, which are often at odds.

Favouring the worst-off implies that patients who run the greatest risk of dying when left untreated, should be treated first (‘sickest first’). It is assumed that other patients, who are less acutely ill, can wait, and be treated later. The principle fits with the ‘rule of rescue’: the psychological imperative to save those who face imminent death [12], even if this comes at disproportionate costs. It follows from the philosophical tradition of prioritarianism, or the notion that it is more valuable to benefit people who are worse off than it is to convey same benefits to people who are better off [13]. However, the ‘sickest first’ principle may lead to the investment of resources in those who have little chance of benefitting [14^▪^], what philosophers call the ‘bottomless pit’ objection. Thus, in the absence of ‘upper limits’ [15], the ‘sickest first’ principle reduces the overall efficiency of the allocation system, and has detrimental effects on the health of the total patient population. This is not in line with the requirements of responsible stewardship [9^▪^] of scarce medical resources: the system should save and improve the lives of patients.

The latter fits with the principle of maximizing total benefits, which follows from utilitarianism, or the notion that ‘doing good’ implies acting in such a way as to bring about as much utility (e.g. health) as possible for all patients, ‘the greatest good for the greatest number’. It aims at saving the most lives (or life-years or quality-adjusted life years). Thus, it would favour the use of posttransplant prognosis or expected medical benefit, not severity, as an allocation principle. The principle of maximizing total benefits thus points in the opposite direction: patients who have the best chances of living a long and healthy life after transplantation should be prioritized for transplantation, and patients with the worst prognosis should be passed over. To many people, however, it seems intuitively unfair to abandon precisely those patients who are most severely ill.

This is the dilemma at the heart of the discussion on the ethics of allocation of donor organs: how can a balance be struck between efficiency and equity, or between utility and justice? The authors of a recent literature review conclude that there is broad support for both parameters – efficiency and equity – but lack of clarity on how to ‘put them into practice’ [8^▪^]. To resolve conflicts between opposing principles, their relative weight or strength must be determined. The authors suggest investigating lay public opinions on this matter [8^▪^].

## A SYNTHESIS OF THE EMPIRICAL EVIDENCE ON PUBLIC PREFERENCES

In recent years, several research groups have investigated the perspectives of stakeholders, including healthcare professionals, patients and lay publics, on the relative importance of equity and efficiency. In an Australian study, a large representative sample of the general population and a group of healthcare professionals were asked to rank allocation principles in order of importance [16]. Among the general population, most highest-ranked principles related to *equity* in allocation, and included needs-based equity, priority for those who had waited the longest, were most difficult to transplant, or were sickest. By contrast, among healthcare professionals, most highest-ranked principles related to *utility*, and included allocating higher-quality organs to those with the best predicted survival or to the young. These findings resonate with previous studies suggesting that lay publics prefer allocation based on need [17]. The authors conclude that policy makers should recognize that there are ‘important differences between priorities held by community members and healthcare professionals working in the transplant field regarding allocation of deceased donor kidneys’ [16].

However, the evidence on public support for equity is limited. A recent German interview study suggests differently: patients and patient representatives prioritized patients with predicted higher life expectancy and, especially, good quality of life after transplantation [18]. Likewise, a study on public preferences for allocation of scarce healthcare services in the UK suggests that the lay public deems ‘save the most lives’ more important than ‘sickest first’ [19]. The authors conclude that ‘people tend to be utilitarians rather than egalitarians’ [19]. A German focus group study found that lay audiences could not choose between the principles of maximizing transplant success and giving priority to urgent patients facing imminent death, and felt that allocation policies should combine the two principles [20]. And a systematic review of the empirical literature confirms this observed difficulty and lack of clarity about how lay publics trade off effectiveness and medical urgency [21]. The evidence is inconclusive. There is no single public preference, and there is little evidence for the claim that general communities and healthcare professionals attach different weights to the principles of equity and utility.

## LIMITATIONS TO THE USE OF PUBLIC PREFERENCES

Even if there *were* public consensus one or more allocation principles, it is not clear how this should guide policy makers in designing allocation systems. What ought to be cannot be derived from what is (the is/ought problem). For instance, empirical studies consistently show that lay publics find that ‘own fault’ should be used as an allocation principle. Patients who use alcohol, tobacco or other substances, decline vaccinations, make other unhealthy lifestyle choices, or have done so in the past, should not qualify for an organ [22]. There is a lifestyle component to many indications for organ transplantation, in liver transplantation, for instance, nonalcoholic steatohepatitis, acute hepatitis B virus infections, acute liver failure after acetaminophen toxicity [23]. Should policy makers heed public preferences to decline allocation to morally ‘undeserving’ [18] recipients?

Recent scholarship considers substance abuse not as a result of flawed character, but of addiction. Addiction is seen a treatable chronic illness [24], caused by an interplay of genetic and nongenetic factors that are largely beyond the control of individual patients. Responsibility for ill health should not be placed on individual patients, and they should not be blamed for unhealthy behaviours by being excluded from organ transplantation. This is not compatible with the ethical principle of equal moral worth. Even if communities might favour a ‘punitive, meritocratic ideology’ [23] in organ allocation, there is no valid ethical rationale for the use of ‘own fault’ as an allocation principle. Luckily, this ideology need not be shared by transplant professionals [25]. At any rate, this analysis illustrates that when public preferences diverge from expert preferences, the former should not always prevail as providing a basis for allocation policy-making.

## HOW TO SOLVE THE DILEMMA?

If the solution of the allocation dilemma does not lie – singularly – in public preferences, balancing and rank-ordering can be used [14^▪^] to make explicit the weighting of prominent ethical values in the justification of allocation policies. In the context of the COVID-19 pandemic, ethicists have recently converged on the primacy of the principle of maximizing benefits or saving the most lives [9^▪^]. Yet if support for maximizing utility is increasing, it must be balanced against justice and fairness requirements. As said, patients should be treated equally, regardless of sex, ethnicity, socioeconomic status, geographical location, etc. Robert Veatch, one of the founding fathers of medical ethics, proposes to rank such deontological principles over consequentialist principles [26]. Thus, formal justice requirements could serve as guardrails in a space that may otherwise be largely guided by utility.

Policies could be evaluated along these lines. For instance, in 2018, the US OPTN modified the donor heart allocation system to improve rates of transplantation for patients at a high risk for waitlist mortality, including patients on short-term mechanical circulatory support [27]. Since the policy change, the use of short-term mechanical circulatory support has increased, but there is centre-level variation in this use. Consequently, the change may have exacerbated inequitable access to heart transplantation along the lines of geographical location [28]. As, at the same time, the new policy has not led to improved posttransplant survival rates [29], it has gained neither in efficiency nor in equity. To this day, geographically determined disparities in access to donor organs persist [30]. Many allocation systems are currently striving for broader geographical sharing of organs across country or regional boundaries [31], with the dual aims of reducing inequitable access and improving efficiency.

And in 2014, the U.S. kidney allocation system was overhauled [32] to gain life years by allocating the best donor organs to the candidates with the highest estimated posttransplant survival, rather than to those who had spent most time on the waiting list. Critics argued that by trying to eliminate longevity mismatch, the programme would lead to age discrimination and worse outcomes for patients with diabetes and presensitized patients [33]. The age discrimination argument can be easily refuted, as older age is not reserved for some, but, rather, something that, if all goes well, all persons will go through. Moreover, according to the ‘fair innings’ argument, younger people should be prioritized over older people, as young people have had less opportunity to experience a normal human lifespan than older patients [34]. This policy may succeed in improving both efficiency and equity.

To facilitate thinking through trade-offs and convergence between efficiency and equity, an American research group has proposed a machine learning approach to modelling the outcomes of various combinations of allocation principles [11]. The approach is meant to help ‘ethicists and community groups’ converge on the ‘right’ balance between utility and fairness and develop or refine policies for the allocation of donor organs.

## BROADENING THE ETHICAL DISCUSSION ON JUSTICE

Although there is relatively much attention in the literature for the fairness of distributing organs among patient on waiting lists for organ transplantation, there is less attention to broader and possibly more serious concerns regarding inequities of access to transplant waiting lists. Patients who may benefit from organ transplantation encounter barriers in referral for transplant evaluation [32]. Patients who are cognitively impaired, frail or suffer from comorbidities, may have smaller chances of being waitlisted [35,36]. Furthermore, there are racial and ethnic disparities in access to transplants, as, for instance, black patients are less likely to be referred for transplant evaluation [37]. Although the aforementioned kidney allocation system policy change in the USA aimed at reducing ethnic and racial disparities by adapting the calculation of waiting time, now beginning at the start of dialysis, it has not fully succeeded [37]. Also, the presence of social support affects patients’ likelihood of being waitlisted [38], but may not be evenly distributed across socioeconomic strata. During or after the COVID-19 pandemic, demonstrating social support may have become more difficult due to travelling and gathering restrictions, especially for more vulnerable patients groups [39]. Also, mandatory COVID-19 vaccination may have exacerbated existing disparities in access to transplantation and transplant waitlisting [40,41]. These justice problems are often hidden from view, as they concern patients who do not achieve being registered on waiting lists. It is high time to start understanding and addressing current disparities in access to transplant waiting lists [42^▪^].

## CONCLUSION

Recent scholarship on the ethics of donor organ allocation suggests that there is no consensus on what constitutes a fair allocation system. There is a particular tension between the allocation principles of favouring the worst-off, which is connected to the value of equity, and of maximizing total benefits, which is connected to the value of utility. The empirical literature on preferences of general publics or stakeholders is inconclusive, and does not help to solve the dilemma. In this study, I have tried to explain the dilemma of the – at times incompatible – demands of justice and utility from an ethical point of view. I suggest that by way of justification, policy makers might try to make explicit – and use new modelling approaches in doing so – how they weigh and rank important ethical values. They are advised to monitor the effects of allocation policy changes on important ethical outcomes, such as the distribution of access to organ transplants across geographical regions, racial or ethnic populations, or socioeconomic strata. Also, I have highlighted recent work suggesting that possibly more serious justice issues related to access to organ transplants, lie not in the space of allocation, but elsewhere, in disparities in referral for transplant evaluation.

## Acknowledgements

*None.*

### Financial support and sponsorship

*None.*

### Conflicts of interest

*There are no conflicts of interest.*

## REFERENCES AND RECOMMENDED READING

Papers of particular interest, published within the annual period of review, have been highlighted as:

▪ of special interest
▪▪ of outstanding interest
